# Gradual Telomere Shortening and Increasing Chromosomal Instability among PanIN Grades and Normal Ductal Epithelia with and without Cancer in the Pancreas

**DOI:** 10.1371/journal.pone.0117575

**Published:** 2015-02-06

**Authors:** Yoko Matsuda, Toshiyuki Ishiwata, Naotaka Izumiyama-Shimomura, Hideki Hamayasu, Mutsunori Fujiwara, Ken-ichiro Tomita, Naoki Hiraishi, Ken-ichi Nakamura, Naoshi Ishikawa, Junko Aida, Kaiyo Takubo, Tomio Arai

**Affiliations:** 1 Department of Pathology, Tokyo Metropolitan Geriatric Hospital, 35-2 Sakae-cho, Itabashi-ku, Tokyo, 173-0015, Japan; 2 Department of Integrated Diagnostic Pathology, Graduate School of Medicine, Nippon Medical School, 1-1-5 Sendagi, Bunkyo-ku, Tokyo, 113-8602, Japan; 3 Research Team for Geriatric Pathology, Tokyo Metropolitan Geriatric Hospital and Institute of Gerontology, 35-2 Sakae-cho, Itabashi-ku, Tokyo, 173-0015, Japan; 4 Department of Pathology, Japanese Red Cross Medical Center, 4-1-22 Hiroo, Shibuya-ku, Tokyo, 150-8935, Japan; 5 Department of Laboratory Medicine, Hadano Red Cross Hospital, Hadano, Kanagawa, 257-0017, Japan; Tulane University Health Sciences Center, UNITED STATES

## Abstract

A large body of evidence supports a key role for telomere dysfunction in carcinogenesis due to the induction of chromosomal instability. To study telomere shortening in precancerous pancreatic lesions, we measured telomere lengths using quantitative fluorescence *in situ* hybridization in the normal pancreatic duct epithelium, pancreatic intraepithelial neoplasias (PanINs), and cancers. The materials employed included surgically resected pancreatic specimens without cancer (n = 33) and with invasive ductal carcinoma (n = 36), as well as control autopsy cases (n = 150). In comparison with normal ducts, telomere length was decreased in PanIN-1, −2 and −3 and cancer. Furthermore, telomeres were shorter in cancer than in PanIN-1 and −2. Telomere length in cancer was not associated with histological type, lesion location, or cancer stage. PanINs with or without cancer showed similar telomere lengths. The incidences of atypical mitosis and anaphase bridges, which are morphological characteristics of chromosomal instability, were negatively correlated with telomere length. The telomeres in normal duct epithelium became shorter with aging, and those in PanINs or cancers were shorter than in age-matched controls, suggesting that telomere shortening occurs even when histological changes are absent. Our data strongly suggest that telomere shortening occurs in the early stages of pancreatic carcinogenesis and progresses with precancerous development. Telomere shortening and chromosomal instability in the duct epithelium might be associated with carcinogenesis of the pancreas. Determination of telomere length in pancreatic ductal lesions may be valuable for accurate detection and risk assessment of pancreatic cancer.

## Introduction

The annual incidence of pancreatic cancer has been increasing worldwide [1], and is a leading cause of cancer-related death [2]. The prognosis of pancreatic cancer remains poor with an overall 5-year survival rate of approximately 5% [1] due to its aggressive growth and high rate of metastasis. Recent studies have shown that pancreatic cancer does not arise de novo, but rather progresses through a multistep process involving non-invasive precursor lesions known as pancreatic intraepithelial neoplasias (PanINs), and culminating in invasive cancer [3,4,5]. Mutations of *KRAS, CDKN2a, TP53*, and *SMAD4*, which are driver mutations in pancreatic cancers, accumulate according to the histological grade of PanINs and drive neoplastic transformation and tumor progression [6,7]. There is a striking link between advanced age and an increased incidence of pancreatic cancer [8,9], and this may represent the combined effects of mutation load, epigenetic regulation, telomere dysfunction, and an altered stromal milieu [10,11].

Telomeres are tandem repeats of the sequence TTAGGG at chromosomal ends in eukaryotes, and play a key role in preventing chromosomal instability [12,13,14]. While telomerase-mediated preservation of telomere function has been shown to promote the development of advanced malignancies [15], there is equally compelling experimental evidence that lack of telomerase activity and a transient period of telomere shortening and dysfunction drive cancer initiation by induction of chromosomal instability [11,16]. Pancreatic cancer is characterized by genomic complexity and instability; telomere shortening, loss of TP53, *K-RAS* mutation, abnormal mitosis and nuclear abnormalities are all contributors to this phenotype [17]. PanIN also harbors chromosomal instability such as telomere shortening [18], aneuploidy [19], loss of heterozygosity [20], and a DNA damage response triggered by activation of the ataxia-telangiectasia-mutated (ATM)-cell cycle checkpoint kinase-2 (Chk2) checkpoint pathway [21]. Telomere shortening appears to precede the development of *TP53* mutations during pancreatic carcinogenesis [18,22,23]. However, any alterations of telomere function during the carcinogenesis step have remained unclear.

Using Southern blotting, we have analyzed the lengths of telomeres in most human organs and tissues, including the pancreatic head, and confirmed that telomeres shorten with age, except for those in cerebral tissue [24,25,26,27,28,29]. The estimated annual reduction rate of telomere length in the pancreas was 36 base pairs [27]. We have also confirmed the telomere length distributions of different cell types in the tongue, esophagus, stomach, breast, skin, and pancreatic islet using quantitative fluorescence *in situ* hybridization (Q-FISH) and our original software, Tissue Telo, employing the telomere: telomere / centromere ratio (TCR) or normalized TCR (NTCR) [30,31,32,33,34,35,36,37,38]. Telomeres in uninvolved epithelium surrounding squamous cell carcinoma *in situ* (CIS) of the tongue and esophagus were shorter than those in age-matched controls [34,39,40].

In the present study, we postulated that pancreatic cancer is likely to arise in duct epithelium with shortened telomeres and chromosomal instability. Using our Q-FISH measurement technique, telomere lengths were estimated in pancreatic duct epithelium with or without cancer, PanINs, and pancreatic cancers. We compared the telomere length of pancreatic duct epithelium between cases showing cancerous change and age-matched control cases. We also histologically estimated the presence of atypical mitosis and anaphase bridges as possible morphological indicators of chromosomal instability [41].

## Materials and Methods

### Patients and tissues

The pancreatic tissues used in this study were obtained from patients who underwent surgical treatment at Tokyo Metropolitan Geriatric Hospital (S1 Table). To analyze telomere length in pancreas specimens without any pathological change, we used autopsy specimens obtained at Tokyo Metropolitan Geriatric Hospital and the Japanese Red Cross Medical Center (S2 Table). These autopsy specimens were employed because of the difficulty in obtaining specimens of normal pancreas unassociated with malignancy or inflammation from surgically resected materials. Although we were concerned that postmortem changes might affect telomere length, we had previously studied telomere lengths in samples of cerebrum and heart obtained after different postmortem intervals using Southern blotting, and found no significant changes among the sampled time points [26]. The present study was conducted in accordance with the principles embodied in the Declaration of Helsinki, 2013, and all experiments were approved by the ethics committees of Tokyo Metropolitan Geriatric Hospital and the Japanese Red Cross Medical Center. Informed written consent for the usage of tissues was obtained from all patients or bereaved families.

### Tissue processing and histological assessment

Tissues were fixed in 10% buffered formalin and then subjected to standard tissue processing and paraffin embedding. The tissues were sliced serially into sections 3 μm thick for hematoxylin and eosin (H&E) staining, and into sections 2 μm thick for Q-FISH. Pathological specimens were diagnosed by our pathologists (YM, HH, JA, TA, and KT) based on the World Health Organization Classification of Tumours of the Digestive System [42]. Samples of the normal pancreatic duct were divided into two groups: normal small duct epithelium (N-small, intercalated duct to intralobular duct), and normal large duct epithelium (N-large, interlobular duct to main pancreatic duct) (S1A Fig.). PanIN lesions were classified as PanIN-1, −2 or −3 [3,4] (S1A Fig.). Many PanINs were found in both surgically resected cases and autopsy cases (S1 Fig. B and C). For analysis of the normal duct and PanINs, we selected areas without inflammation, as inflammation is known to influence telomere length [43].

### Quantitative fluorescence *in situ* hybridization (Q-FISH) for analysis of telomeres

The slides were processed by the FISH method, as reported previously [30,31,32,33,39]. Tissue sections were hybridized with PNA probes for the telomere (telo C-Cy3 probe, '5-CCCTAACCCTAACCCTAA-3'; Fasmac, Kanagawa, Japan) and the centromere (Cenp1-FITC probe, '5-CTTCGTTGGAAACGGGGT-3'; Fasmac), and the nuclei were stained with DAPI (Molecular Probes, Eugene, OR, USA).

FISH images were captured by a CCD camera (ORCA-ER-1394, Hamamatsu Photonics KK, Hamamatsu, Japan) mounted on a microscope (80i, Nikon, Tokyo, Japan). Microscope control and image acquisition were performed using the Image-Pro Plus software package (version 5.0, Media Cybernetics Co. Ltd., Silver Spring, MD, USA). The captured images were analyzed using our own software, ‘TissueTelo Ver. 3.1’, which estimates the TCRs of individual nuclei, as reported previously [30,31,32,33,44]. As there is no guarantee that the entire nucleus will be captured within any given tissue section, the total corrected telomere signal for each nucleus is normalized by the corresponding integrated optimal density of the centromere [30,32]. TCR values were determined from individual cells of N-small, N-large, PanIN and cancer, based on the histological findings of serial H&E-stained sections. Over 100 cells (mean 187) were analyzed for each sample. We obtained adequate FISH data for 66 N-small, 61 N-large, 42 PanIN-1, 27 PanIN-2, 15 PanIN-3, and 34 cancer samples from surgically resected specimens and 150 N-large samples from autopsy specimens.

As a control for variations in sample preparation, we also performed Q-FISH on sections of a block preparation of a cultured cell strain, TIG-1 (34 PDL: telomere length, 8.57 kbp by Southern blot analysis)[45], and placed them on the same slides as the pancreatic sections. The TCR measurement for each pancreatic cell was divided by the median TCR for the control cell block on the same slide to give the NTCR of the cell [30,31].

### Analysis of atypical mitotic figures and anaphase bridges

Metaphase figures were observed at ×400 magnification using representative H&E slides by two of our pathologists (YM and JA). Pyknotic nuclei or nuclei with basophilic cytoplasm were not considered as mitosis. An atypical mitotic figure was defined anything other than the typical form of normal mitosis, including multipolar mitosis, ring mitosis, dispersed mitosis, asymmetrical mitosis and lag-type mitosis [46]. An anaphase bridge was defined as a filamentous connection linking two well-separated and parallel-aligned groups of anaphase chromosomes [41]. We counted the numbers of mitoses, atypical mitoses and anaphase bridges per 1,000 nuclei in the normal duct epithelium, PanIN and cancer, and calculated the respective percentages of total mitoses, atypical mitoses and anaphase bridges. The total mitosis count included atypical mitoses and anaphase bridges.

### Ethics statement

This study was conducted in accordance with the principles embodied in the Declaration of Helsinki, 2008, and written informed consent for the usage of tissues was obtained from all patients or their bereaved families. Experiments were approved by the Ethics Committee of Tokyo Metropolitan Geriatric Hospital and Institute of Gerontology (permit-#260219).

### Statistical analysis

Differences between two groups were analyzed using Student’s *t* test or Mann-Whitney U test. Differences among multiple groups were analyzed using post hoc test. The chi-squared test was used to analyze clinicopathological features. The level of significance was set at *P<0*.05 for all analyses. Based on TCR, receiver-operator characteristic (ROC) analyses were performed, and the area under the curve (AUC) value was determined. Statistical analyses were performed using the StatView J version 5.0 software package (SAS Institute, Inc., Cary, NC, USA) and XLSTAT (Addinsoft, New York, NY, USA).

## Results

### Telomere shortening in pancreatic precancerous lesions

**Fig 1:**
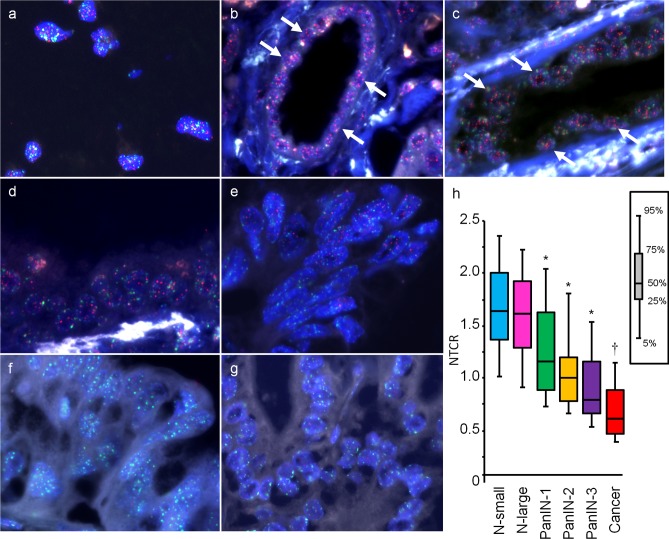
FISH images of the TIG-1 cell line and pancreatic ductal lesions. Original magnification, x400. Red, telomere-Cy3 signal; green, centromere-FITC signal; blue, DAPI counterstaining for DNA. (a) Q-FISH image of the control TIG-1 cell block placed on the same slide together with pancreas sections. (b-g) Q-FISH images of normal small duct (N-small) (b), normal large duct (N-large) (c), PanIN-1 (d), PanIN-2 (e), PanIN-3 (f), and pancreatic invasive ductal adenocarcinoma (g). Telomere signals were evident in normal small duct (b, arrows) and normal large duct (c, arrows), while telomere signals decreased from PanIN-1 to PanIN-3 based on visual assessment (d, e and f). Telomere signals were barely visible in cancer cells (g). (h) Box-and-whisker plot of the NTCR for each of the pancreatic ductal lesions surgically resected from patients with pancreatic cancer (n = 36) and without pancreatic cancer (n = 33). *P<0.*05* vs N-small and N-large. †*P<0*.*05* vs N-small, N-large, PanIN-1 and −2.

As a control in Q-FISH analysis, a TIG-1 cell block was placed on the same slide together with each pancreas specimen (Fig. 1A). Telomeres (red) were evident in the nuclei of the normal duct (Fig. 1B and C, arrows), while PanIN-1, −2 and −3, and cancer showed weak telomere signals (Fig. 1D, E, F and G). We determined the TCR of each lesion using the Tissue Telo software package. The distributions of telomere intensity indicated by TCR showed a decrease of telomere length in PanINs and cancers in comparison with the normal duct (S2 Fig. A and B). As compared with the normal duct (N-small and N-large), NTCR values for PanIN-1, −2, and −3 and cancer were significantly decreased (Fig. 1H, *P<0*.05). Furthermore, NTCR values for cancer were lower than those for PanIN-1 and −2 (Fig. 1H, *P<0*.05). There was no significant difference between small and large duct epithelium. The NTCR for normal ducts and PanINs with and without cancers also showed no significant differences (S3 Fig., gray bar vs white bar). The NTCR for pancreatic cancers showed no correlation with tumor stage, location or histological classification (S3 Table).

### Telomere length and chromosomal instability

**Fig 2:**
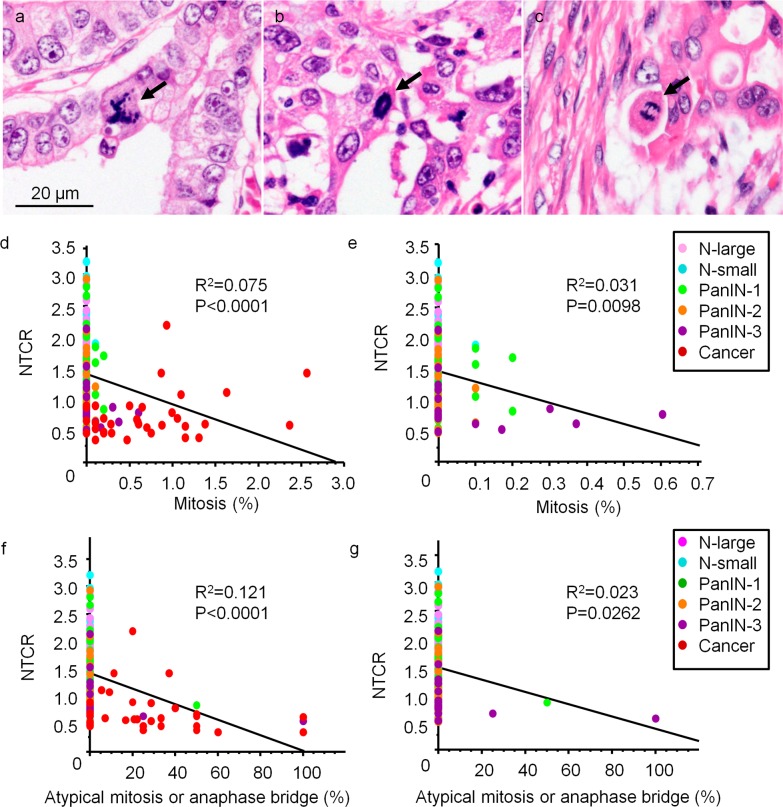
Relationship between telomere length and presence of atypical mitotic figures including anaphase bridges. (a-c) Representative atypical mitotic figures. Arrows indicate multipolar mitosis (a), ring mitosis (b), and an anaphase bridge (c). (d and e) Correlation between NTCR and mitosis for the lesions as a whole (d) and overall minus cancer (e). Data are shown as the percentage of total mitotic Figures among the total cell count. (f and g) Correlation between NTCR and percentage of atypical mitoses or anaphase bridges among the lesions as a whole (f) and overall minus cancer (g). Data are shown as the percentage of atypical mitoses or anaphase bridges among the total number of mitotic figures.

Atypical mitoses including multipolar mitoses (Fig. 2A) and ring mitoses (Fig. 2B), as well as anaphase bridges (Fig. 2C), were observed in pancreatic cancers. Mitoses were very few in the normal duct, while PanIN and cancer showed an increased number of mitoses (Table 1, *P<0.05, **P<0.*01*). Atypical mitoses and anaphase bridges were not detected in the normal duct, but were increased in cancer (Table 1, *P<0.05, **P<0.*01*). Pancreatic cancers had a mitotic index of 0.7%, and 26% of mitoses were atypical or involved anaphase bridges.

**Table 1:** Mitosis and anaphase bridges in pancreatic tissues.

		Number of positive lesions		Mean value	
	Number of lesions	Mitosis	Atypical mitosis or anaphase bridge	Mitosis/cell (%)	(Atypical mitosis or anaphase bridge)/mitosis (%)
N-small	66	1	0	0	0
N-large	61	0	0	0	0
PanIN-1	42	5 *	1	0.02	1.19
PanIN-2	27	4 *	0	0.01	0
PanIN-3	15	6 *	2	0.10	8.33
Cancer	34	31 *	26 *	0.70 **	25.79 **

N-small, intercalated duct to intralobular duct; N-large, interlobular duct to main pancreatic duct; PanIN, pancreatic intraepithelial neoplasia; Cancer, pancreatic invasive ductal adenocarcinoma.

*P<0.05 vs N-small and N-large by chi-squared test.

**P<0.01 vs other lesions by post-hoc test.

We found a negative correlation between the NTCR and the incidence of mitoses (Fig. 2D, all lesions, *P<0*.*0001*, R^2^ = 0.075; Fig. 2E, lesions except for cancers, *P = 0*.*0098*, R^2^ = 0.031). The incidence of atypical mitoses or anaphase bridges also showed a negative correlation with NTCR (Fig. 2F, all lesions, *P<0*.*0001*, R^2^ = 0.121; Fig. 2G, lesions except for cancers, *P = 0*.*0262*, R^2^ = 0.023).

### Telomere shortening in non-cancerous duct epithelium

**Fig 3:**
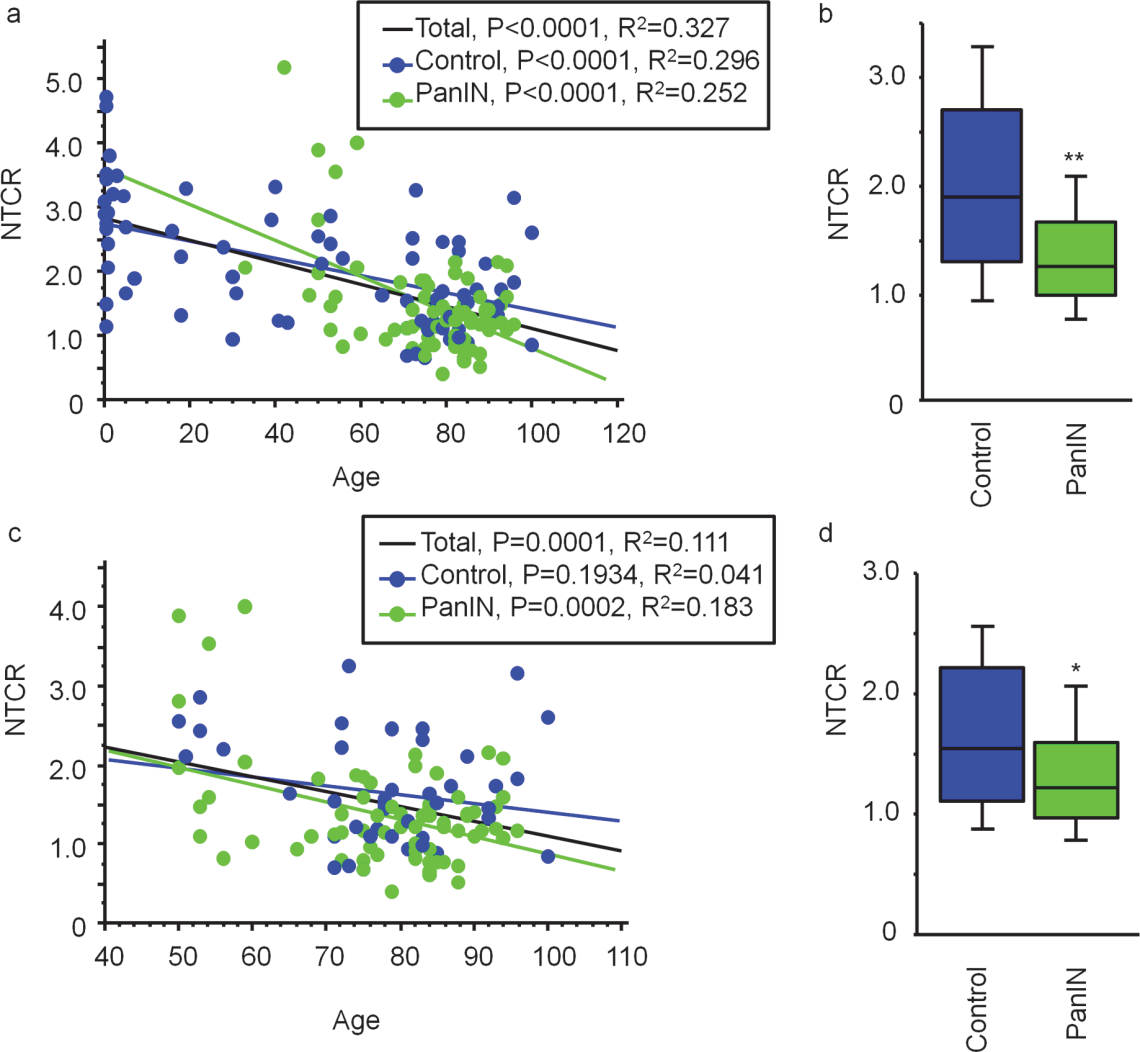
Telomere length in normal duct epithelium from autopsy cases. (a) NTCR in the normal duct epithelium in 150 autopsy cases was negatively correlated with age for the cases overall (n = 150, black), the control group (n = 77, blue) and the PanIN group (n = 73, green). (b) Comparison of NTCR in the normal duct epithelium between the control and PanIN groups. **P<0.*01* vs control. (c) NTCR in normal duct epithelium from individuals aged over 50 years (n = 113). Blue, control (n = 43); green, PanIN group (n = 70). (d) Comparison of NTCR in the normal duct epithelium between the control and PanIN groups. *P<0.*05* vs control.

To assess alterations of telomeres in cells without histological change, we analyzed telomere length in non-cancerous duct epithelium (N-large) using autopsy specimens. The NTCR for normal duct epithelium was negatively correlated with increased age (Fig. 3A, black line, *P<0*.*0001*, R^2^ = 0.327). We divided the autopsy cases into two groups, i.e. those with and without PanINs, and the patients with PanIN were older than control patients (S2 Table, ***P<0.*0001*). In the control and PanIN groups, there was a negative correlation between NTCR and age (Fig. 3A; control, blue line, *P<0*.*0001*, R^2^ = 0.296; PanIN, green line, *P<0*.*0001*, R^2^ = 0.252). The NTCR for the normal duct epithelium was reduced by 29% in the PanIN group relative to the control group (Fig. 3B, **P<0.*01* vs control). Age-matched analyses revealed a negative correlation between the NTCR for normal duct epithelium and age (Fig. 3C, black line, *P = 0*.*0001*, R^2^ = 0.111). In the PanIN group there was a negative correlation between NTCR for the normal duct epithelium and age (Fig. 3C; green line, *P = 0*.*0002*, R^2^ = 0.183), whereas this relationship was not statistically significant in the control group (Fig. 3C; blue line, *P = 0*.*1934*, R^2^ = 0.041). The NTCR for the normal duct epithelium was reduced by 16% in the PanIN group relative to age-matched controls (Fig. 3D, *P<0.*05* vs control).

**Fig 4:**
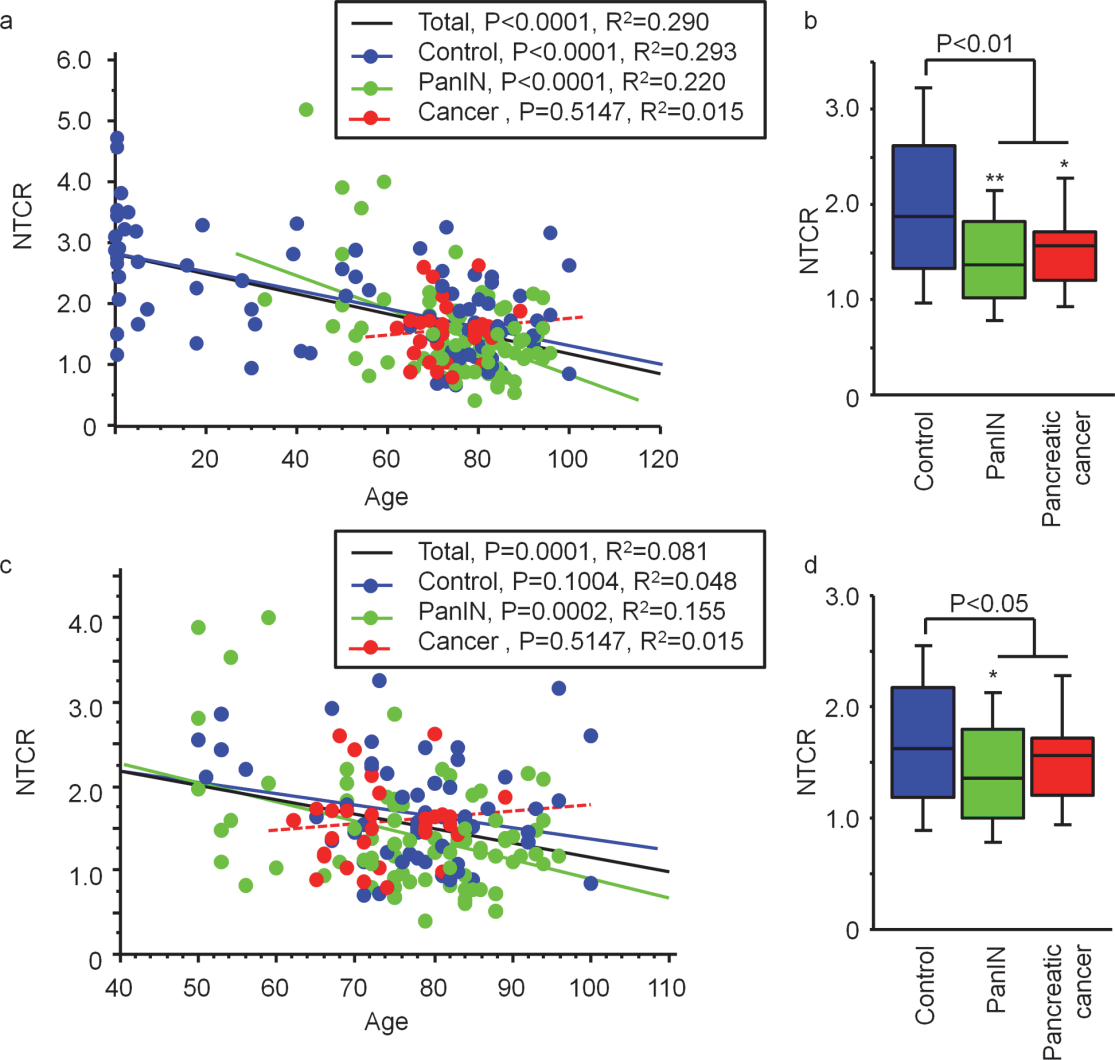
Telomere length in normal duct epithelium from autopsy and surgically resected cases. (a) NTCR in the normal duct epithelium in 150 autopsy and 69 surgically resected cases was negatively correlated with age in the cases overall (n = 219, black), the control group (n = 91, blue) and the PanIN group (n = 92, green), but the relationship was not statistically significant in the pancreatic cancer group (n = 36, red). (b) Comparison of NTCR in the normal duct epithelium between the control, PanIN and pancreatic cancer groups. *P<0.*05* vs control. **P<0.*01* vs control. (c) NTCR in the normal duct epithelium from individuals aged over 50 years (n = 182). Blue, control (n = 57); green, PanIN (n = 189); red, pancreatic cancer group (n = 36). (d) Comparison of NTCR in the normal duct epithelium among the control, PanIN and pancreatic cancer groups. *P<0.*05* vs control.

For aggregate analyses of autopsy and surgically resected cases, we divided the cases into three groups: controls (without PanIN and pancreatic cancer), cases with PanIN, and cases with pancreatic cancer. The NTCR for the normal duct epithelium showed a negative correlation with age (Fig. 4A; black line, *P<0*.*0001*, R^2^ = 0.290). In the control and PanIN groups, NTCR was negatively correlated with age (Fig. 4A; control, blue line, *P<0*.*0001*, R^2^ = 0.293; PanIN, green line, *P<0*.*0001*, R^2^ = 0.220). In the pancreatic cancer group, the relationship between age and NTCR was not statistically significant (Fig. 4A, red line, *P = 0*.*5147*, R^2^ = 0.015). In the PanIN group and the pancreatic cancer group, the NTCR was reduced by 26% and 23%, respectively, relative to the control group (Fig. 4B, *P<0.*05*, **P<0.*01* vs control). Age-matched analyses demonstrated a negative correlation between the NTCR for the normal duct epithelium and age (Fig. 4C, black line, *P = 0*.*0001*, R^2^ = 0.081). The PanIN groups also showed a negative correlation between NTCR and age (Fig. 4C, green line, *P = 0*.*0002*, R^2^ = 0.155), whereas no such correlation was evident in the control and pancreatic cancer groups (Fig. 4C; control, blue line, *P = 0*.*1004*, R^2^ = 0.048; pancreatic cancer, red line, *P = 0*.*5147*, R^2^ = 0.015). The NTCR was reduced by 15% in the PanIN group and by 9% in the pancreatic cancer group relative to the age-matched controls (Fig. 4D, *P<0.*05* vs control).

## Discussion

The present study revealed that telomeres in histologically normal duct epithelium associated with PanINs or cancers were shorter than those in normal duct epithelium unassociated with PanINs or cancers, and that telomeres shortened progressively from PanIN-1 to PanIN-3. Aging, along with the increased incidence of PanINs, was also correlated with marked shortening of telomeres in the normal duct epithelium. These results indicate that in the elderly, the duct epithelium, which harbors shortened telomeres, may give rise to PanINs and pancreatic cancers. Furthermore, as compared to age-matched controls, the telomeres in histologically normal duct epithelium were shortened in individuals with PanINs or cancers, suggesting that factors other than age might influence telomere length. Previously, we have reported that the background epithelium of CIS in the esophagus, tongue, and skin has shorter telomeres and more chromosomal instability than control tissue [34,36,40]. Taken together, critical telomere dysfunction in the background, possibly due to higher annual telomere attrition, might give rise to chromosomal instability and carcinogenesis in the pancreatic ductal epithelium.

A previous study using FISH analysis has shown that telomere shortening was nearly universal in all histological grades of PanIN [18]. In that study, Heek et al. determined telomere length in 10 nuclei from each lesion, and therefore heterogeneity of the telomere length distribution might have influenced their results. In contrast, we determined telomere length in over 100 nuclei from each lesion, and found an inverse correlation between telomere length and PanIN histological grade. Our results suggest that telomere shortening in noninvasive lesions of the pancreatic duct induce accumulation of chromosomal abnormalities, leading to the development of invasive ductal adenocarcinomas.

Both cell proliferation and telomerase activity influence telomere shortening. In the present study, the mitotic index was negatively correlated with telomere length, reflecting the fact that telomeres become shortened every time a cell divides. A higher rate of cellular proliferation due to inflammation and subsequent tissue regeneration might lead to more rapid telomere shortening. However, we did not analyze the relationship between telomerase activity, cell growth ratio and telomere length because the use of formalin-fixed paraffin-embedded tissues made this impossible.

In pancreatic cancers, we also identified high incidences of anaphase bridges and atypical mitotic figures, accounting for roughly a quarter of all mitoses. A previous study of pancreatic cancer cell lines showed that anaphase bridges were detectable in 14–50% of anaphase cells in association with the bridge-breakage-fusion cycle induced by telomeric dysfunction [18]. Malignant tumors appear to represent a heterogeneous group that may be categorized on the basis of both chromosomal instability and the telomere maintenance mechanism. Among rectal cancers, those with chromosomal instability show shortened telomeres and telomerase activation, whereas those with chromosomal stability show lengthening of telomeres without shortening and telomerase activation [47]. In the present study, all of the invasive pancreatic ductal adenocarcinomas showed markedly shortened telomeres. A previous report has indicated that acinar-to-ductal metaplasia coexisting with PanIN also exhibits telomere shortening; therefore acinar-to-ductal metaplasia has been considered a precursor lesion of pancreatic cancer [48]. Taken together, the available data suggest that telomere-dependent chromosomal instability may have a significant role in pancreatic carcinogenesis.

Our present findings suggest that determination of telomere length in cells obtained by biopsy or in samples of pancreatic juice may be of value for both detection and risk assessment of pancreatic cancer. ROC analyses for distinction between pancreatic cancer lesions (including PanIN-3 and cancers) and non-cancerous tissue (including normal duct, PanIN-1 and −2) using NTCR revealed the usefulness of telomere length as an indicator of cancer susceptibility (S4 Fig.). The AUC for NTCR was 0.869, being similar to that of serum CA19-9 [49,50].

Pancreatic neuroendocrine tumors often show altered lengthening of telomeres (ALT), which are detected as larger spots of telomere signals [51], but in the present study, no larger spots of telomere signals were evident in the pancreatic duct and cancers. Although our Q-FISH method is highly accurate and reproducible, it has several drawbacks: (1) The presence of telomere fusion cannot be assessed. (2) Any abnormality of the centromere might influence the telomere/centromere ratio. (3) As with other FISH methods, it is not possible to detect telomere and centromere signals if the fixation methods or other sample preparation techniques are unsuitable.

In conclusion, telomere shortening occurs in the pancreatic ductal epithelium in the early stage of carcinogenesis in the absence of any evident histological changes. Critically shortened telomeres in the pancreatic duct epithelium and in noninvasive ductal lesions lead to accumulation of chromosomal abnormalities, and in turn to the development of pancreatic invasive ductal adenocarcinomas via PanINs. Determination of telomere length in pancreatic ductal lesions may be of value for accurate detection and risk assessment of pancreatic cancer.

## Supporting Information

S1 Fig Pancreatic duct, noninvasive ductal lesions and invasive ductal adenocarcinomas. (a) Representative images of N-small (intralobular duct, arrows), N-large (interlobular ducts, arrows), PanIN-1, −2, and −3, and pancreatic cancer. (b and c) Incidence of PanIN by age in surgically resected (b, n = 36) and autopsy (c, n = 150) cases of pancreatic cancer. PanIN-1 became more common with increasing age, whereas PanIN-3 was not found in cases without pancreatic cancer.
(TIF)

S2 Fig Distributions of telomere intensity expressed as TCR in TIG-1 cells, the pancreatic duct, and ductal lesions. TCR tended to be lower in PanIN and cancer lesions than in the normal duct epithelium (N-small and N-large) in a case of pancreatic cancer (a) and in a case without pancreatic cancer (b).
(TIF)

S3 Fig Comparison of NTCR in various types of tissue between pancreatic cancer cases and controls. There were no statistically significant differences in NTCR between cancer cases (gray bar) and controls (white bar). ND, no difference.
(TIF)

S4 Fig ROC curve analysis of NTCR for detection of pancreatic cancer. NTCR for surgically resected cases was used in ROC analysis for distinction between pancreatic cancer (including cancer and PanIN-3) and non-cancerous duct (including normal duct and PanIN-1 and −2).
(TIF)

S1 Table Characteristics and incidence of PanINs in surgically resected cases. (DOCX)

S2 Table Characteristics and incidence of PanINs in autopsy cases. (DOCX)

S3 Table Telomere length and clinicopathological characteristics of surgically resected pancreatic cancer cases. (DOCX)
