# Supplementary figures and images for: Gradual Telomere Shortening and Increasing Chromosomal Instability among PanIN Grades and Normal Ductal Epithelia with and without Cancer in the Pancreas
Source: PLoS One. 2015 Feb 6;10(2):e0117575. doi: 10.1371/journal.pone.0117575 (PMC4319908; doi:10.1371/journal.pone.0117575)

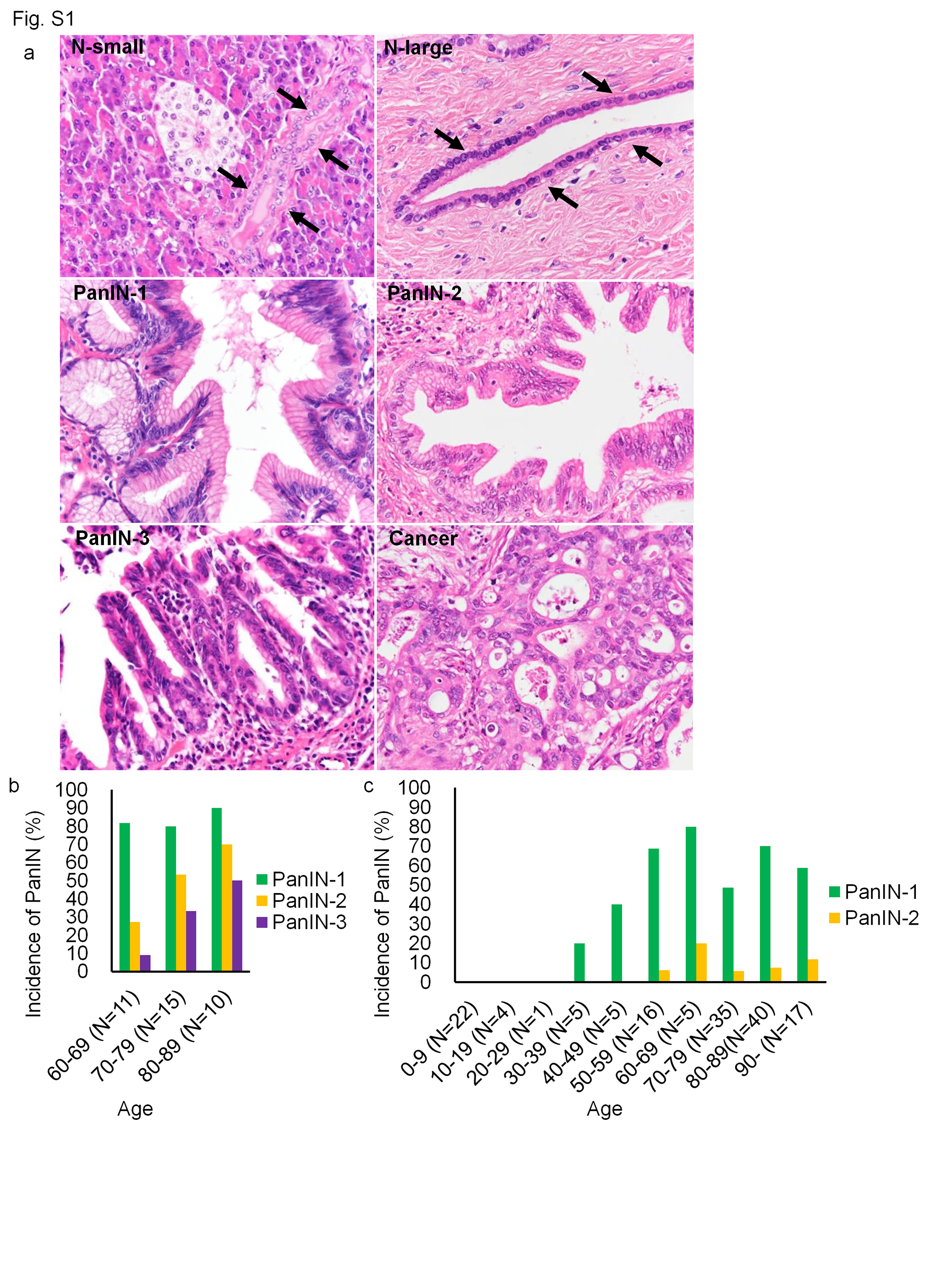

Supplement: S1 Fig — (a) Representative images of N-small (intralobular duct, arrows), N-large (interlobular ducts, arrows), PanIN-1, −2, and −3, and pancreatic cancer. (b and c) Incidence of PanIN by age in surgically resected (b, n = 36) and autopsy (c, n = 150) cases of pancreatic cancer. PanIN-1 became more common with increasing age, whereas PanIN-3 was not found in cases without pancreatic cancer. (TIF) [file pone.0117575.s001.tif]

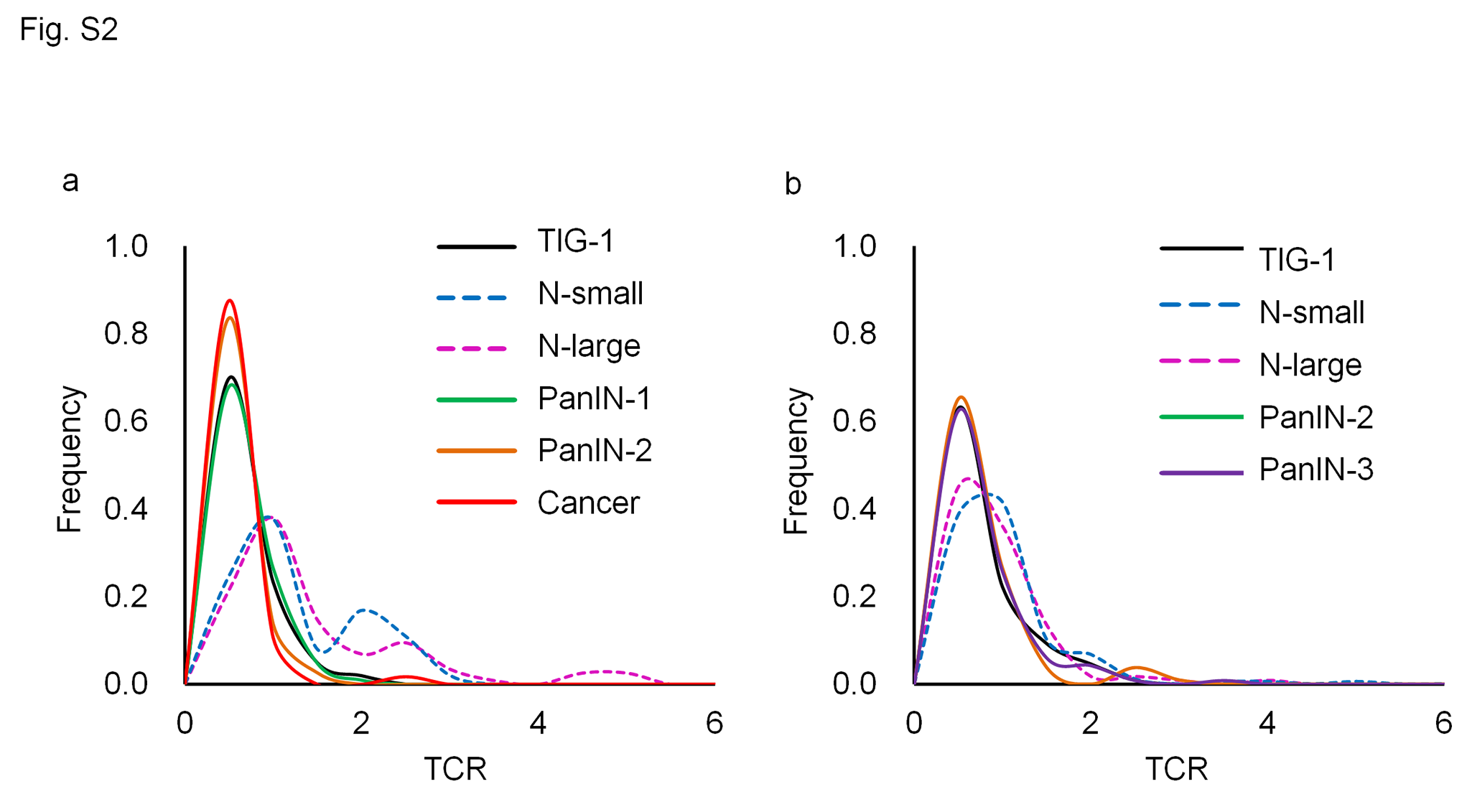

Supplement: S2 Fig — TCR tended to be lower in PanIN and cancer lesions than in the normal duct epithelium (N-small and N-large) in a case of pancreatic cancer (a) and in a case without pancreatic cancer (b). (TIF) [file pone.0117575.s002.tif]

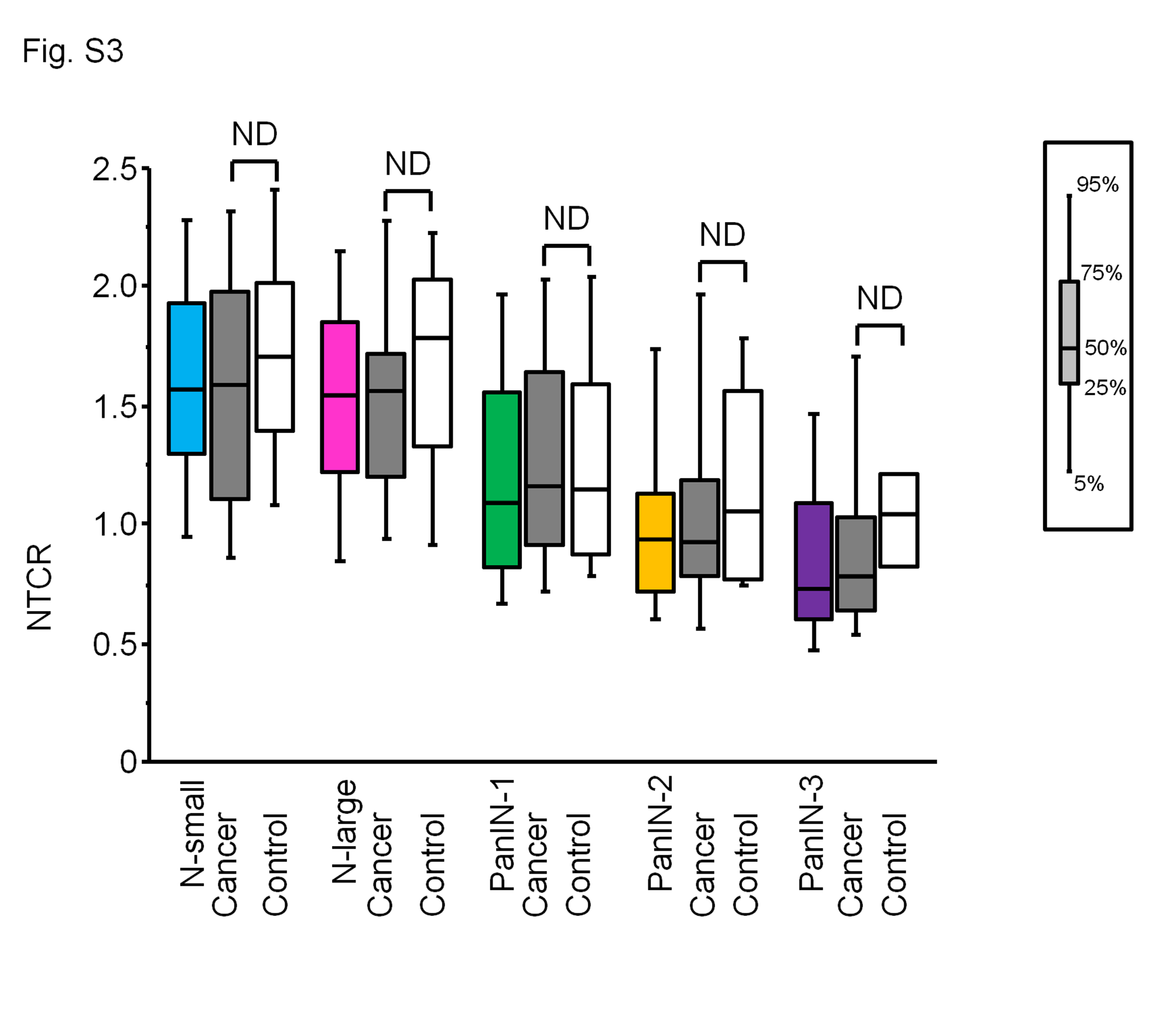

Supplement: S3 Fig — There were no statistically significant differences in NTCR between cancer cases (gray bar) and controls (white bar). ND, no difference. (TIF) [file pone.0117575.s003.tif]

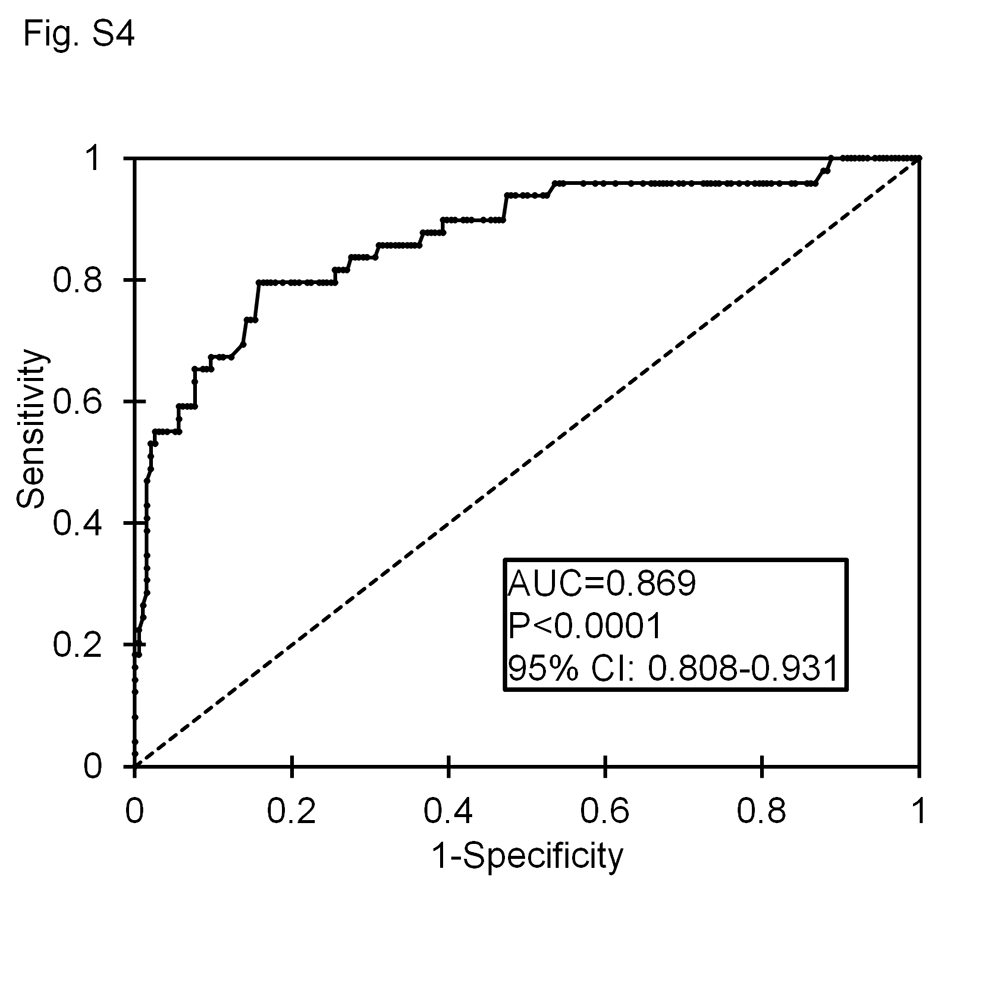

Supplement: S4 Fig — NTCR for surgically resected cases was used in ROC analysis for distinction between pancreatic cancer (including cancer and PanIN-3) and non-cancerous duct (including normal duct and PanIN-1 and −2). (TIF) [file pone.0117575.s004.tif]
