# Supplementary material for: Gradual Telomere Shortening and Increasing Chromosomal Instability among PanIN Grades and Normal Ductal Epithelia with and without Cancer in the Pancreas
Source: PLoS One. 2015 Feb 6;10(2):e0117575. doi: 10.1371/journal.pone.0117575 (PMC4319908; doi:10.1371/journal.pone.0117575)
Supplement: S1 Table — (DOCX) [file pone.0117575.s005.docx]

| **Table S1. Surgically resected cases** | | | | | |  | | | |  | |  | | | | | | |  |
| --- | --- | --- | --- | --- | --- | --- | --- | --- | --- | --- | --- | --- | --- | --- | --- | --- | --- | --- | --- |
|  |  | Total cases | | | Control cases‡ | | | | | Pancreatic cancer cases† | | | | | | | | |  |
| Number | | 69 | | | 33 | | | | | 36 | | | | | | | | |  |
| Age | | 74.87 ± 6.34 | | | 76.00 ± 5.90 | | | | | 73.83 ± 6.63 | | | | | | | | |  |
| Sex | |  |  | |  | |  | | | |  | |  | | |  | | |  |
|  | Male | 30 | | | 18 | | | | | 12 | | | | | | |  |  |  |
|  | Female | 39 | | | 15 | | | | | 24 | | | | | | |  |  |  |
| PanIN | |  |  | |  | | |  | | |  | | |  | | | |  | |
|  | Cases without PanIN | 22 | (31.88) | | 15 | | | (45.45) | | | 7 | | | (19.44) | | | |  | |
|  | PanIN-1 | 47 | (68.12) | | 18 | | | (54.55) | | | 29 | | | (80.56) | | | | * | |
|  | PanIN-2 | 29 | (42.03) | | 10 | | | (30.30) | | | 19 | | | (52.78) | | | |  | |
|  | PanIN-3 | 15 | (21.74) | | 3 | | | (9.09) | | | 12 | | | (33.33) | | | | * | |
| Brackets indicate percentages. | | |  | |  | | |  | | |  | | |  | | | |  | |
| ‡Cases without pancreatic cancers. Patients underwent surgical treatment for heterotopic spleen, lymphoma, or carcinoma of the stomach, Vater papilla, gallbladder, or bile duct. | | | | | | | | | | | | | | | | |  |  |  |
| †Cases of pancreatic invasive ductal adenocarcinoma. | | | | | |  | | |  | | |  | | |  | |  |  |  |
| *P<0.05 vs control by chi-squared test. | | | |  | |  | | |  | | |  | | |  | |  |  |  |
