# Supplementary material for: Gradual Telomere Shortening and Increasing Chromosomal Instability among PanIN Grades and Normal Ductal Epithelia with and without Cancer in the Pancreas
Source: PLoS One. 2015 Feb 6;10(2):e0117575. doi: 10.1371/journal.pone.0117575 (PMC4319908; doi:10.1371/journal.pone.0117575)
Supplement: S2 Table — (DOCX) [file pone.0117575.s006.docx]

| **Table S2. Autopsy cases** | | | |  |  |  |
| --- | --- | --- | --- | --- | --- | --- |
|  |  | Total cases | | Control cases‡ | PanIN cases† |  |
| Number | | 150 | | 77 | 73 |  |
| Age | | 61.99 ± 30.77 | | 48.62 ± 35.92 | 76.10 ± 14.30 | *** |
| Sex | |  |  |  |  |  |
|  | Male | 83 | | 44 | 39 |  |
|  | Female | 66 | | 32 | 34 |  |
|  | Undetermined | 1 | | 1 | 0 |  |
| PanIN | |  |  |  |  |  |
|  | Cases without PanIN | 77 | (51.33) | 77 | 0 |  |
|  | PanIN-1 | 73 | (48.67) | 0 | 73 |  |
|  | PanIN-2 | 9 | (6.00) | 0 | 9 |  |
|  | PanIN-3 | 0 |  | 0 | 0 |  |
| Brackets indicate percentages. | | |  |  |  |  |
| ‡Cases without pancreatic intraepithelial neoplasia (PanIN). | | | | |  |  |
| †Cases with PanIN-1, -2 or -3. | | |  |  |  |  |
| ***P<0.0001 vs control cases. | | |  |  |  |  |
