# Supplementary material for: Gradual Telomere Shortening and Increasing Chromosomal Instability among PanIN Grades and Normal Ductal Epithelia with and without Cancer in the Pancreas
Source: PLoS One. 2015 Feb 6;10(2):e0117575. doi: 10.1371/journal.pone.0117575 (PMC4319908; doi:10.1371/journal.pone.0117575)
Supplement: S3 Table — (DOCX) [file pone.0117575.s007.docx]

| **Table S3. Telomere length and clinicopathological characteristics of surgically resected pancreatic cancer cases** | | | | | |
| --- | --- | --- | --- | --- | --- |
|  |  | Number | NTCR‡ | | |
| Age | |  | |  |  |
|  | <74 | 18 | 0.75 | ± | 0.43 |
|  | ≥74 | 18 | 0.71 | ± | 0.32 |
| Sex | |  |  |  |  |
|  | Male | 12 | 0.77 | ± | 0.29 |
|  | Female | 24 | 0.71 | ± | 0.41 |
| Location | |  |  |  |  |
|  | Pancreatic head | 27 | 0.79 | ± | 0.40 |
|  | Pancreatic body | 5 | 0.50 | ± | 0.12 |
|  | Pancreatic tail | 4 | 0.60 | ± | 0.18 |
| Stage† | |  |  |  |  |
|  | I | 2 | 0.95 | ± | 0.69 |
|  | II | 32 | 0.72 | ± | 0.37 |
|  | IV | 2 | 0.62 | ± | 0.04 |
| Histological type | |  |  |  |  |
|  | Well differentiated adenocarcinoma | 17 | 0.77 | ± | 0.34 |
|  | Moderately differentiated adenocarcinoma | 15 | 0.70 | ± | 0.46 |
|  | Asenosquamous carcinoma | 4 | 0.66 | ± | 0.18 |
| ‡Normalized telomere / centromere ratio | |  |  |  |  |
| †Union for International Cancer Control | |  |  |  |  |
